# Atrial cardiomyopathy as a multidomain disease: longitudinal evidence for autonomic remodelling

**DOI:** 10.1093/europace/euag132

**Published:** 2026-06-30

**Authors:** Jean-Baptiste Guichard, David Hupin, Vincent Pichot, Sébastien Celle, Inés Martínez Saludes, Ahmed El-Medany, Joseph Barker, Ivo Roca-Luque, Lluís Mont, Antoine Da Costa, Adelina Doltra, Fu Siong Ng, Eduard Guasch, Linda S Johnson, Frédéric Roche

**Affiliations:** Institut Clínic Cardiovascular (ICCV), Hospital Clínic, Universitat de Barcelona, C/ Villarroel 170, 08036 Barcelona, Catalonia, Spain; Institut D’Investigacions Biomèdiques August Pi I Sunyer (IDIBAPS), Villarroel 170, 08036 Barcelona, Catalonia, Spain; National Heart and Lung Institute, Hammersmith Campus, Imperial College London, Du Cane Road, London W12 0NN, UK; Department of Clinical and Exercise Physiology, University of Jean Monnet, University Hospital of Saint-Etienne, Mines Saint-Etienne, INSERM, U 1059, Saint-Etienne, Saint-Priest-en-Jarez 42270, France; Department of Clinical and Exercise Physiology, University of Jean Monnet, University Hospital of Saint-Etienne, Mines Saint-Etienne, INSERM, U 1059, Saint-Etienne, Saint-Priest-en-Jarez 42270, France; Department of Clinical and Exercise Physiology, University of Jean Monnet, University Hospital of Saint-Etienne, Mines Saint-Etienne, INSERM, U 1059, Saint-Etienne, Saint-Priest-en-Jarez 42270, France; Institut Clínic Cardiovascular (ICCV), Hospital Clínic, Universitat de Barcelona, C/ Villarroel 170, 08036 Barcelona, Catalonia, Spain; National Heart and Lung Institute, Hammersmith Campus, Imperial College London, Du Cane Road, London W12 0NN, UK; National Heart and Lung Institute, Hammersmith Campus, Imperial College London, Du Cane Road, London W12 0NN, UK; Institut Clínic Cardiovascular (ICCV), Hospital Clínic, Universitat de Barcelona, C/ Villarroel 170, 08036 Barcelona, Catalonia, Spain; Institut D’Investigacions Biomèdiques August Pi I Sunyer (IDIBAPS), Villarroel 170, 08036 Barcelona, Catalonia, Spain; Institut Clínic Cardiovascular (ICCV), Hospital Clínic, Universitat de Barcelona, C/ Villarroel 170, 08036 Barcelona, Catalonia, Spain; Institut D’Investigacions Biomèdiques August Pi I Sunyer (IDIBAPS), Villarroel 170, 08036 Barcelona, Catalonia, Spain; Department of Clinical and Exercise Physiology, University of Jean Monnet, University Hospital of Saint-Etienne, Mines Saint-Etienne, INSERM, U 1059, Saint-Etienne, Saint-Priest-en-Jarez 42270, France; Cardiology Department, University Hospital of Saint-Étienne, Saint-Étienne, France; Institut Clínic Cardiovascular (ICCV), Hospital Clínic, Universitat de Barcelona, C/ Villarroel 170, 08036 Barcelona, Catalonia, Spain; Institut D’Investigacions Biomèdiques August Pi I Sunyer (IDIBAPS), Villarroel 170, 08036 Barcelona, Catalonia, Spain; National Heart and Lung Institute, Hammersmith Campus, Imperial College London, Du Cane Road, London W12 0NN, UK; Department of Cardiology, Hammersmith Hospital, Imperial College Healthcare NHS Trust, London, UK; Department of Cardiology, Chelsea and Westminster Hospital NHS Foundation Trust, London, UK; Institut Clínic Cardiovascular (ICCV), Hospital Clínic, Universitat de Barcelona, C/ Villarroel 170, 08036 Barcelona, Catalonia, Spain; Institut D’Investigacions Biomèdiques August Pi I Sunyer (IDIBAPS), Villarroel 170, 08036 Barcelona, Catalonia, Spain; Lund University, Department of Clinical Sciences, Lund, Sweden; Department of Clinical and Exercise Physiology, University of Jean Monnet, University Hospital of Saint-Etienne, Mines Saint-Etienne, INSERM, U 1059, Saint-Etienne, Saint-Priest-en-Jarez 42270, France; Cardiology Department, University Hospital of Saint-Étienne, Saint-Étienne, France

**Keywords:** Atrial cardiomyopathy, Atrial fibrillation, Autonomic remodelling, Heart rate variability, Risk stratification

## Abstract

**Aims:**

Atrial cardiomyopathy (AtCM) is increasingly recognized as a substrate for atrial fibrillation (AF), yet its operationalization remains limited and largely marker-driven. Whether autonomic remodelling represents a longitudinally evolving domain within a multidomain framework of AtCM remains unclear.

**Objectives:**

To determine whether autonomic remodelling, assessed through static and longitudinal heart rate variability (HRV) abnormalities, defines a distinct domain of AtCM and contributes to atrial disease burden and clinical risk.

**Methods and results:**

We studied 670 individuals aged 65 years without prior AF or major cardiovascular disease from the prospective PROOF cohort with 24-h Holter ECGs at baseline and 5 years. Heart rate variability metrics, premature atrial contraction (PAC) burden, and left atrial (LA) size were assessed. Incident AF and cardiovascular outcomes were adjudicated over a median follow-up of 12.1 years. Seventy-two participants (10.7%) developed AF. Static HRV abnormalities and adverse 5-year HRV trajectories were independently associated with subsequent AF. Autonomic abnormalities showed limited concordance with PAC burden and LA enlargement, supporting their role as a distinct AtCM domain. Increasing involvement of remodelling domains was associated with higher risks of AF and cardiovascular outcomes. Participants with ≥2 domains exhibited higher risks of AF (HR 2.43; 95% CI 1.72–3.45), major adverse cardiovascular events (HR 1.42), and all-cause mortality (HR 1.35).

**Conclusion:**

Atrial cardiomyopathy is a cumulative, multidomain disease process in which structural, electrical, and autonomic abnormalities define atrial disease burden. Longitudinal autonomic remodelling constitutes an independent and evolving axis within this framework, shifting the focus from isolated arrhythmia detection towards progressive characterization of atrial substrate.

## Graphical Abstract

**Graphical Abstract:**
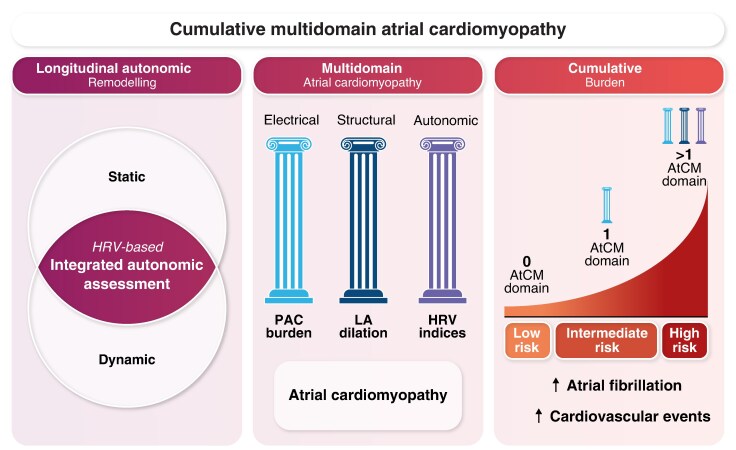
Cumulative Multidomain Atrial Cardiomyopathy Static and longitudinal autonomic measures capture dynamic autonomic remodelling. Integrated with electrical and structural abnormalities, they define a cumulative multidomain atrial cardiomyopathy in which increasing domain burden is associated with higher risks of atrial fibrillation and cardiovascular events.

What Is new?

- Atrial cardiomyopathy is demonstrated as a cumulative multidomain disease integrating structural, electrical, and autonomic remodelling.
- Longitudinal autonomic changes, assessed through heart rate variability trajectories, identify a dynamic and independent axis of atrial disease progression.
- The integration of autonomic abnormalities significantly improves risk stratification for incident atrial fibrillation beyond conventional markers.

What are the clinical implications?

- Atrial fibrillation should be considered a late manifestation of a progressive atrial disease rather than an isolated arrhythmia.
- Multidomain assessment combining structural, electrical, and autonomic markers may enable earlier identification of patients at risk before irreversible remodelling occurs.
- Longitudinal autonomic profiling using repeated heart rate variability measurements could support dynamic risk stratification and future prevention strategies.

## Introduction

Atrial fibrillation (AF) often becomes clinically apparent only after substantial and frequently irreversible atrial remodelling has occurred and may first manifest as AF-related stroke.^1^ Despite advances in rhythm detection, current AF paradigms remain predominantly rhythm-centric, focusing on arrhythmia identification rather than the underlying progression of atrial disease. Early identification of individuals with subclinical atrial disease therefore remains a major unmet need in cardiovascular prevention.^2^

The concept of atrial cardiomyopathy (AtCM), encompassing structural, functional, and electrical atrial abnormalities, has emerged as a framework to characterize subclinical atrial disease before overt arrhythmia.^3^ However, current definitions remain heterogeneous and largely marker-driven, limiting reproducibility across studies and hindering clinical translation. Structural enlargement and electrical markers alone may not fully capture the multidimensional nature of atrial remodelling, which likely reflects the cumulative interaction of multiple biological processes driving atrial disease progression.^4^

Autonomic dysregulation represents one potential but underrecognized component of this process.^5^ The atria are densely innervated, and alterations in autonomic balance may contribute to electrophysiological and structural remodelling. Heart rate variability (HRV), a noninvasive marker of cardiac autonomic modulation, has been associated with incident AF,^6^ stroke,^7,8^ and cardiovascular mortality.^9^ However, most studies rely on static HRV indices, which capture only a physiological snapshot and may not adequately reflect the dynamic nature of atrial disease progression. Whether autonomic remodelling represents a distinct AtCM domain in the general population—and whether its longitudinal evolution contributes to a cumulative, multidomain framework of atrial disease—remains uncertain.^10,11^

We therefore hypothesized that autonomic remodelling constitutes a distinct and dynamic AtCM domain. By integrating baseline autonomic markers with their longitudinal trajectories, we aimed to refine AtCM as a cumulative multidomain disease process and to better characterize atrial disease progression beyond conventional structural and electrical markers.

## Methods

### Study population

**Figure 1:**
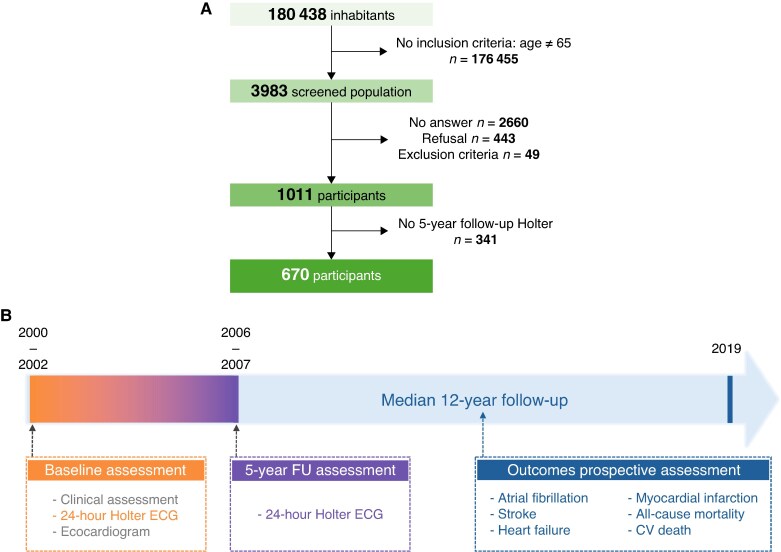
Study design and longitudinal follow-up framework in the PROOF cohort. **Panel A.** Participant flow from the PROOF cohort. Of 1011 individuals enrolled at age 65 years, 670 had complete baseline and 5-year Holter ECG data and were included in the analysis. **Panel B.** Timeline of HRV assessment and outcome follow-up. HRV was measured at baseline and at 5 years using 24-h Holter ECGs, allowing evaluation of static HRV indices and longitudinal HRV changes. Clinical outcomes—including incident AF (primary outcome), stroke, myocardial infarction, heart failure, cardiovascular death, and all-cause mortality—were prospectively adjudicated over a median follow-up of 12.1 years from baseline. AF, atrial fibrillation; CV, cardiovascular; ECG, electrocardiogram; FU, follow-up; T0, baseline; T5, 5-year follow-up.

The PROOF study (NCT00759304) is a population-based prospective cohort designed to evaluate autonomic nervous system (ANS) activity as a predictor of cardiovascular and cerebrovascular outcomes in older adults.^12^ It enrolled an age-homogeneous population of 65-year-olds, thereby eliminating chronological age as a confounder. Between September 2000 and December 2002, all 65-year-old residents of Saint-Étienne, France, were invited to participate (***Figure 1****A*). Individuals with prior AF, myocardial infarction, stroke, heart failure, cardiac implantable electronic devices, and terminal illness (life expectancy <5 years) or residence in long-term care facilities were excluded (*n* = 49). Of 3983 invitees, 1011 (25.4%) were enrolled.

Although the overall PROOF cohort has a median follow-up of 17.8 years from baseline, the present analysis was restricted to participants with technically adequate 24-h Holter recordings at both baseline (T0) and the 5-year visit (T5). Time-to-event analyses were conducted using a landmark design with time zero defined at T5, yielding a median follow-up of 12.1 years (IQR 11.2–13.0). Participants without a 5-year Holter were not included, and those who developed AF before T5 were excluded from analyses involving longitudinal HRV change to preserve temporal sequence. No participants were lost to follow-up. The study conforms to the Declaration of Helsinki; all participants provided written informed consent, and the protocol was approved by the local ethics committee.

### Heart rate variability and atrial cardiomyopathy assessment

As illustrated in ***Figure 1****B*, autonomic activity was measured at baseline (T_0_) and at the 5-year follow-up (T_5_) using HRV parameters derived from 24-h Holter ECG recordings (StrataScan 563, Del Mar Avionics, Irvine, CA). Baseline HRV (T0) was retained as the static autonomic marker to capture early autonomic status preceding structural and electrical atrial remodelling, while event-free survival was assessed from T5 to ensure temporal separation between longitudinal HRV change and outcome occurrence. RR intervals were extracted, and artefacts and premature beats were corrected by cubic–spline interpolation.^13^ Heart rate variability indices were computed in accordance with ESC/ACC-AHA guidelines.^13^ HRV metrics were computed using a custom-developed,^14^ open-access software tool, ensuring full transparency and reproducibility of the analyses. Standard measures included HRV time– and frequency–domain indices (detailed in **Supplementary material**). Advanced indices included heart fragmentation markers—the percentage of inflection point (PIP) and percentage of short segment (PSS)—as well as non-linear measures (short-term fractal scaling exponent α1)^15^ and acceleration/deceleration capacity (AC/DC).^16^ Clinical covariates are described in the **Supplementary material**. Clinical covariates in the prediction model included sex, hypertension, diabetes, and vascular disease. Atrial cardiomyopathy markers were systematically assessed: Premature atrial complexes (PACs) burden > 500/24h^17^ on baseline Holter ECG (***Figure 1****B*), and left atrial (LA) enlargement, defined by area >24 cm^2^ on the apical four- and two-chamber view and/or an indexed volume >34 mL/m^2^ on baseline transthoracic echocardiography.^18^

### Assessment of clinical outcomes

Incident AF was prospectively adjudicated per international guideline definitions.^19^ Twenty-four-hour Holter recordings were performed at baseline and at 5, 9, and 12 years. Any suspected AF was confirmed by 12-lead ECG or validated single-lead tracings.^6^ When AF was diagnosed by a general practitioner, ECG tracings were collected to verify the diagnosis. Cardiovascular events were captured during research centre visits and from hospital records and physician questionnaires. All-cause and cardiovascular death were verified by registry data and certificate review. Major adverse cardiovascular events (MACE) were defined as a composite of cardiovascular death, myocardial infarction, heart failure hospitalization, or stroke.^20,21^

### Data availability

The data underlying this study are available from the corresponding author upon reasonable request. Public deposition of the dataset was not possible due to institutional and patient confidentiality restrictions.

### Statistical analysis 

Continuous variables were presented as median (IQR) or counts and percentages. Longitudinal changes in HRV between baseline and 5-year follow-up were assessed using paired parametric or nonparametric tests, as appropriate. For dynamic analyses, a landmark approach was applied with time zero defined at the 5-year visit; participants who developed AF before T_5_ were excluded to avoid reverse causality. Associations between HRV and incident AF were evaluated using Cox proportional hazards models adjusted for sex and mean Holter heart rate, selected *a priori* based on physiological relevance and to avoid overadjustment. The proportional hazards assumption was assessed using Schoenfeld residuals and was not violated. Linearity was evaluated using natural splines, and effect estimates were expressed using clinically interpretable contrasts. Static and dynamic autonomic abnormality constructs were derived from multivariable associations and dichotomized using ROC-derived thresholds with bootstrap validation. Sensitivity analyses were performed by excluding participants receiving rate-limiting or antiarrhythmic medications to assess the robustness of autonomic associations. Supportive analyses evaluated whether autonomic abnormalities added prognostic information beyond conventional AtCM markers using Kaplan–Meier analyses, Cox models, and nested models. Analyses were performed in R 4.5.0. Detailed methodological procedures are provided in the Supplementary Methods.

## Results

### Study population and follow-up

Of the 1011 participants enrolled in the PROOF cohort, 670 met inclusion criteria and had technically adequate 24-h Holter recordings at both baseline and the 5-year visit (***Figure 1****A* and *B*). Early attrition within the first 3 years explains a substantial part of the incomplete 5-year Holter data. Baseline characteristics were similar between included and non-included participants (see Supplementary material online, *Table S1*). The median age was 65.1 years (IQR: 65.0–66.1), and 41.2% were men. Hypertension was present in 35.8% of participants and diabetes in 5.7% (***Table 1***). At baseline, conventional markers of AtCM were present in a substantial proportion of the cohort: 9.0% had a high PAC burden (>500 PACs/24 h), 23.9% had LA dilation, and the median CHA_2_DS_2_-VA score was 2 (IQR: 1–2).

**Table 1:** Baseline clinical characteristics and atrial cardiomyopathy features of the study population

***Clinical features***		
Age	65.09	65.01–66.12
Male gender	276	41.19%
Diabetes mellitus	38	5.67%
Hypertension	240	35.82%
Dyslipidaemia	250	37.37%
Thyroid disorders	103	15.37%
COPD	30	4.51%
History of tobacco use	235	35.18%
Obesity	63	9.40%
Severe OSA	86	12.84%
***Baseline use of medications affecting HRV***		
β-Blockers	56	8.36%
Non-dihydropyridine CCB	12	1.79%
***Features characterizing atrial cardiomyopathy***		
PAC burden (per hour)	2.71	1.22–6.21
Increased PAC burden	60	8.96%
Dilated left atrium	116	23.86%
CHADS VA score	2	1–2

Baseline characteristics of participants included in the analysis. Continuous variables are presented as median (interquartile range) and categorical variables as counts and percentages. Features characterizing AtCM are organized according to electrical remodelling (premature atrial contraction burden), structural remodelling (left atrial dilatation), and overall cardiovascular risk burden (CHADS-VA score). Autonomic markers were evaluated separately using heart rate variability metrics.

CHADS-VA, congestive heart failure, hypertension, age ≥75, diabetes mellitus, stroke/transient ischaemic attack, vascular disease, age 65–74; COPD, chronic obstructive pulmonary disease; OSA, obstructive sleep apnoea; PAC, premature atrial contraction.

Over a median follow-up of 12.1 years (IQR = 11.2–13.0), 72 participants (10.7%) developed incident AF, corresponding to an incidence rate of 5.9 per 1000 person-years (95% CI: 4.6–7.1). During follow-up, 66 participants (9.9%) experienced stroke, 144 (21.5%) had MACE, 41 (6.1%) died from cardiovascular causes, and 99 (14.8%) died from any cause.

### Static autonomic domain and incident atrial fibrillation

**Figure 2:**
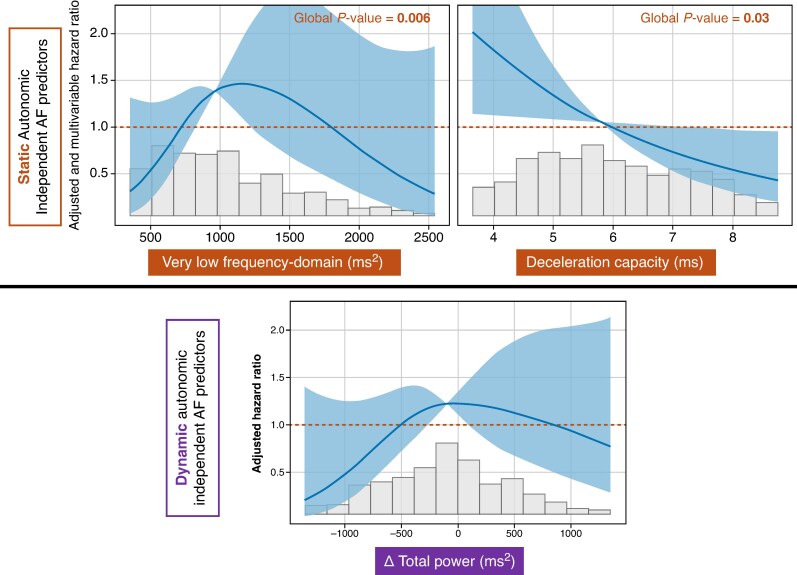
Static and dynamic autonomic markers associated with incident atrial fibrillation. **Panel A.** Static autonomic markers. Associations between baseline VLF power and deceleration capacity and incident AF. **Panel B.** Dynamic autonomic marker. Association between 5-year change in total power (Δ total power) and incident AF. Adjusted hazard ratios (HRs) were estimated using multivariable Cox proportional hazards models with natural cubic splines. Solid lines represent adjusted HR estimates and shaded areas 95% confidence intervals. The dashed horizontal line denotes HR = 1. Histograms depict the distribution of each marker. Estimates are shown at a fixed covariate profile (median age, modal sex, and median Holter mean heart rate). AF, atrial fibrillation; HR, hazard ratio; HRV, heart rate variability; VLF, very low frequency.

Baseline autonomic markers were first evaluated as continuous variables using Cox proportional hazards models adjusted for sex, and Holter mean heart rate, allowing for potential non-linear effects through natural cubic splines. Several markers were associated with incident AF, with formal likelihood–ratio testing identifying non-linearity for selected frequency–domain indices (see Supplementary material online, *Table S2*). After multivariable adjustment including forced clinical covariates and one representative HRV marker per autonomic domain, selected based on the strongest adjusted association, two baseline autonomic predictors remained independently associated with AF. Very low frequency (VLF) power demonstrated a significant non-linear association with AF risk (global *P* = 0.006; *P* for non-linearity = 0.004), characterized by a bell-shaped (inverted U-shaped) relationship, with higher AF risk observed at both low and high VLF values. These independently retained markers are shown in ***Figure 2****A* and were subsequently summarized into a baseline autonomic abnormality construct. To translate these findings into a clinically interpretable construct, a continuous static autonomic abnormality score derived from the multivariable model was dichotomized using ROC-derived thresholds to facilitate clinical interpretation (see Supplementary material online, *Table S3*). Individuals classified as having static autonomic abnormalities experienced a markedly higher risk of incident AF, with clear separation of Kaplan–Meier curves and a hazard ratio of 4.47 (95% CI 2.45–8.14) over a 12-year follow-up (see Supplementary material online, *Figure S1*). These associations remained consistent after exclusion of participants receiving rate-limiting or antiarrhythmic medications (HR 5.03, 95% CI 2.72–9.31), supporting the robustness of the findings.

### Longitudinal changes in HRV and dynamic associations with incident atrial fibrillation

Over a median interval of 4.9 years (IQR: 4.4–5.2), HRV parameters showed significant within-individual changes (***Table 2***). Time–domain indices remained largely stable, with modest increases in parasympathetic-related markers (RMSSD and pNN50). In contrast, frequency–domain measures declined, driven by a marked reduction in VLF power and smaller decreases in total power, low-frequency (LF) power, and the LF/HF ratio, whereas high-frequency (HF) power was unchanged. Advanced metrics evolved in parallel, with increased heart rate fragmentation and concomitant declines in non-linear dynamics (α_1_) and baroreflex-related markers (most comparisons *P* < 0.001).

**Table 2:** Five-year longitudinal changes in heart rate variability metrics

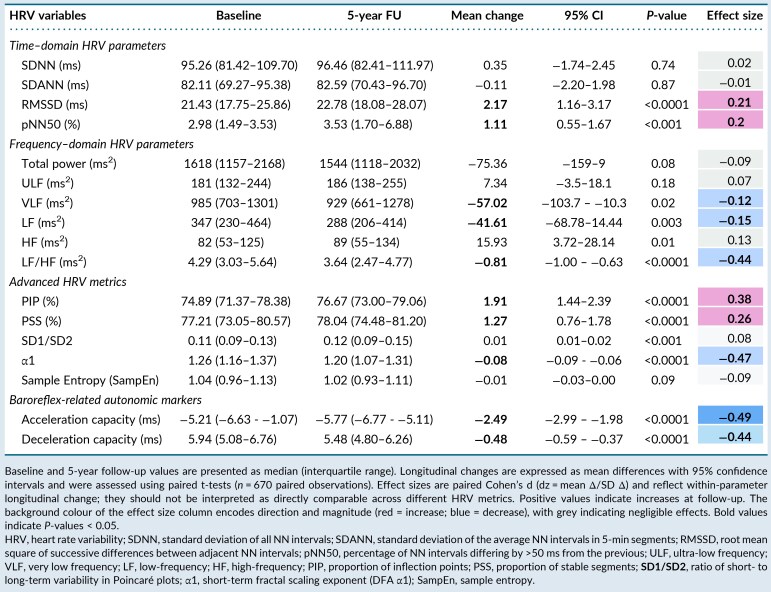

The prognostic significance of these longitudinal changes was evaluated using Cox models adjusted for sex and change in mean heart rate (see Supplementary material online, *Table S4*). Dynamic associations with incident AF were confined to the frequency domain. Change in total power demonstrated a significant non-linear association with AF risk (global *P* = 0.03; *P* for non-linearity = 0.03), characterized by a bell-shaped (inverted U-shaped) pattern, with increased AF risk at both marked decreases and marked increases in total power (***Figure 2****B*). Given that the dynamic signal was restricted to a single autonomic domain and to preserve model parsimony relative to the number of events, a multidomain multivariable dynamic model was not constructed. A dynamic autonomic abnormality construct derived from this association showed moderate discrimination (AUC = 0.67), stable after bootstrap validation (see Supplementary material online, *Table S5*), and identified individuals at higher risk of AF (HR 3.26, 95% CI 1.83–5.82; Supplementary material online, *Figure S2*). Similar results were observed after exclusion of participants receiving rate-limiting or antiarrhythmic medications (HR 2.86, 95% CI 1.55–5.29), confirming the robustness of the dynamic association.

### Integrated static and longitudinal autonomic remodelling

**Figure 3:**
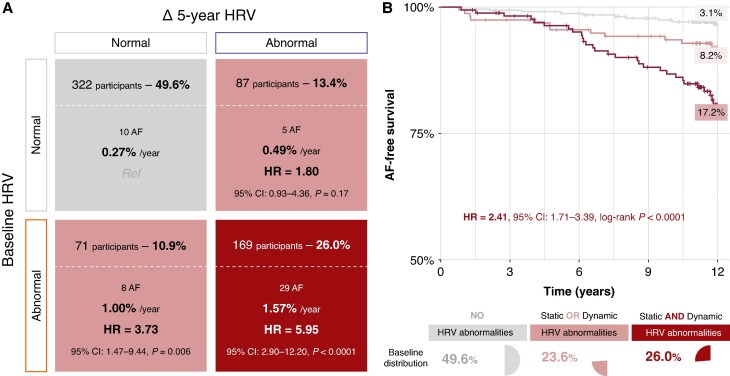
**Static and dynamic HRV abnormalities and incident atrial fibrillation. Panel A.** Cumulative incidence, incidence rates, and hazard ratios for AF across four groups defined by baseline (static) HRV status and 5-year HRV change (dynamic). **Panel B.** Kaplan–Meier curves for AF-free survival according to cumulative HRV abnormality burden, categorized as no abnormalities, isolated static or dynamic abnormalities, and combined static and dynamic abnormalities. The baseline distribution of participants across groups is shown below the x-axis. AF, atrial fibrillation; HR, hazard ratio; HRV, heart rate variability; CI, confidence interval.

Participants were stratified according to baseline autonomic status and 5-year HRV change ((***Figure 3****A*). Individuals without autonomic abnormalities showed a low AF incidence, whereas the presence of static autonomic abnormalities was associated with a higher AF risk. When summarized as a three-level autonomic burden construct integrating static and dynamic abnormalities, AF-free survival showed early and sustained separation across groups (***Figure 3****B*). Over 12 years of follow-up, cumulative AF incidence increased stepwise with autonomic burden, reaching approximately 17% in participants with combined static and dynamic abnormalities (HR 2.41, 95% CI 1.71–3.39; log-rank *P* < 0.0001 per increasing abnormality).

**Figure 4:**
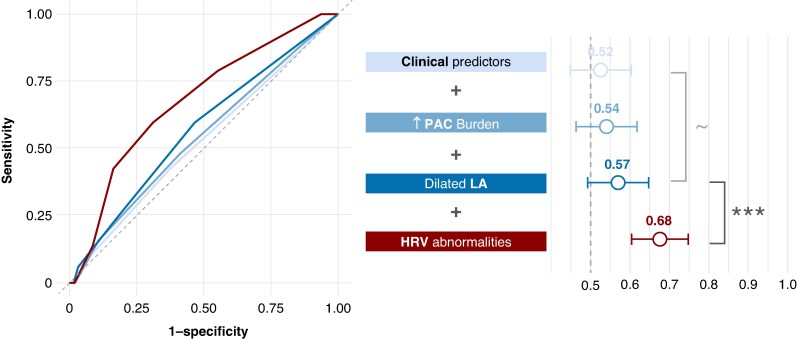
**Contribution of autonomic abnormalities within the multidomain atrial cardiomyopathy framework. Panel A.** Receiver operating characteristic (ROC) curves for sequential models evaluating incident AF across atrial cardiomyopathy domains. **Panel B.** Area under the ROC curve (AUC) with 95% confidence intervals for each model. Models were sequentially constructed by adding premature atrial contraction burden, left atrial dilatation, and HRV abnormalities to a baseline clinical model. AF, atrial fibrillation; AUC, area under the curve; CI, confidence interval; HRV, heart rate variability; PAC, premature atrial contraction. *P < 0.05; **P < 0.01.

Autonomic abnormalities showed limited overlap with established AtCM markers, with weak correlations with clinical risk (CHA_2_DS_2_-VASc score; Spearman ρ = 0.166, *P* < 0.0001) and LA dilatation (ρ = 0.196, *P* < 0.0001) and no association with premature atrial contraction (PAC) burden (see Supplementary material online, *Figure S3*). In hierarchical models evaluating AtCM domains (***Figure 4***), discrimination was poor for the clinical model alone (AUC 0.53) and improved modestly after addition of electrical and structural markers. Inclusion of autonomic abnormalities was associated with a marked improvement in discrimination (AUC 0.68; 95% CI 0.61–0.75 ; *P* < 0.001), with significant improvements in reclassification and overall model fit (NRI, IDI, and likelihood–ratio testing; Supplementary material online, *Table S6*).

### Stratification by atrial cardiomyopathy severity and association with clinical outcomes

**Figure 5:**
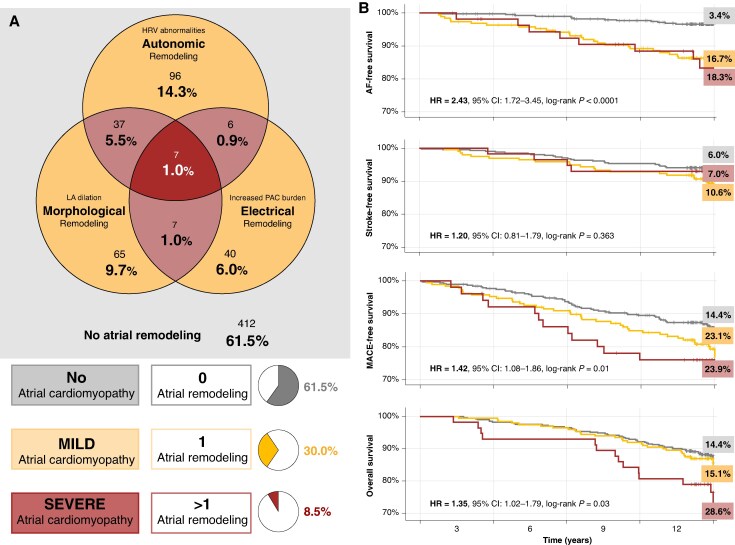
**Multidimensional atrial cardiomyopathy: phenotypic overlap and association with adverse clinical outcomes. Panel A.** Distribution of participants according to the presence of electrical (PAC burden), structural (left atrial dilatation), and autonomic (HRV abnormalities) atrial remodelling domains. **Panel B.** Kaplan–Meier curves for AF, stroke, major adverse cardiovascular events, and all-cause mortality stratified by the number of atrial cardiomyopathy domains (0, 1, or ≥2). AF, atrial fibrillation; HRV, heart rate variability; LA, left atrium; MACE, major adverse cardiovascular events; PAC, premature atrial contraction.

Participants were classified into no, mild, or severe AtCM, defined by the presence of 0, 1, or ≥2 atrial remodelling domains across electrical, structural, and autonomic axes, respectively (***Figure 5****A*). Overall, 61.5% of participants had no evidence of atrial remodelling, 30.0% exhibited involvement of a single domain, and 8.5% fulfilled criteria for severe AtCM. Overlap between remodelling domains was limited, with only 1.0% of individuals exhibiting abnormalities across all three domains. Kaplan–Meier analyses demonstrated a graded association between AtCM severity and incident AF (***Figure 5****B*), with individuals classified as having severe AtCM experiencing a more than two-fold higher risk compared with those without AtCM (HR = 2.43; 95% CI: 1.72–3.45).

Stroke incidence did not differ significantly across AtCM categories, with wide confidence intervals around the effect estimate (HR = 1.29; 95% CI: 0.81–1.79). Beyond arrhythmic outcomes, severe AtCM was associated with higher rates of MACE (HR = 1.42; 95% CI: 1.08–1.86) and all-cause mortality (HR = 1.35; 95% CI: 1.02–1.79).

## Discussion

### Main findings

In this population-based cohort, our findings support AtCM as a multidomain disease process in which autonomic remodelling represents a distinct and longitudinally evolving domain. Static and longitudinal HRV abnormalities were associated with incident AF, suggesting that autonomic perturbation reflects early atrial substrate vulnerability. Increasing involvement of remodelling domains was associated with higher risks of AF, cardiovascular events, and mortality beyond structural and electrical markers.

### Multidomain and cumulative remodelling in atrial cardiomyopathy

Atrial cardiomyopathy lacks a unified clinical definition,^3^ limiting its integration into practice.^2^ Structural and electrical markers alone do not capture the complexity of atrial remodelling. In our cohort of individuals without prior AF or major cardiovascular disease, structural, electrical, and autonomic domains showed limited concordance,^22^ indicating that no single marker adequately reflects atrial disease. Subclinical AtCM was highly prevalent, suggesting that atrial remodelling begins before clinically apparent AF. The cumulative involvement of remodelling domains showed a graded association with AF, cardiovascular events, and mortality, supporting a multidimensional approach to atrial disease characterization.^23,24^ Incorporating longitudinal autonomic trajectories extends AtCM from a static construct to a dynamic disease process, refining identification of progressive atrial remodelling before overt arrhythmia.

### Autonomic remodelling as a dynamic disease process

Atrial fibrillation should be interpreted as a continuum of atrial disease rather than a binary rhythm classification.^25^ Our findings identify autonomic remodelling as an upstream and dynamic component of AtCM, not captured by conventional AF pattern definitions. Although HRV is not anatomically specific to the atrium, it reflects integrated cardiac autonomic regulation.^5^ In our cohort, both short-term HRV metrics and fragmentation indices were associated with incident AF, suggesting that altered variability reflects disorganized autonomic signalling rather than physiological vagal tone in this age group.^26–28^ Importantly, longitudinal HRV trajectories were independently associated with AF, indicating that evolving autonomic imbalance mirrors progressive atrial substrate remodelling. Participants with both baseline and dynamic abnormalities showed early divergence in AF-free survival. These findings support a model in which autonomic remodelling represents an independent and evolving axis of AtCM, with implications extending beyond AF to broader cardiovascular risk.^29–32^

### Clinical perspectives

These findings support conceptualizing AtCM as a cumulative, multidomain disease process rather than a condition defined by isolated abnormalities. Integrating structural, electrical, and autonomic domains may enable more comprehensive characterization of atrial disease burden and earlier identification of preclinical states before irreversible remodelling occurs.

Within this framework, longitudinal autonomic profiling may reflect evolving atrial health rather than merely predict arrhythmic events. The growing feasibility of repeated HRV assessment through wearable technologies may facilitate such phenotyping in selected populations.^33^ These findings also support moving beyond a binary definition of AF towards a burden-based framework.^34^ Atrial fibrillation burden likely reflects the underlying atrial substrate more accurately than a dichotomous classification, with autonomic remodelling contributing to both AF occurrence and its temporal expression.

Collectively, these findings support viewing AtCM as a cumulative, multidomain, and longitudinal disease process that precedes overt arrhythmia and shapes long-term cardiovascular risk.

### Study limitations

Participation represented approximately one-quarter of eligible individuals, which may introduce selection bias, although baseline characteristics were comparable to the source population. The monocentric design enabled highly standardized assessments and long-term follow-up but may limit generalizability. The homogeneous age cohort reduces confounding related to age-dependent autonomic variability but may limit extrapolation to more heterogeneous populations. Heart rate variability reflects global autonomic regulation; however, within a continuum-based model of AF,^25^ it captures a relevant systemic component of atrial disease. Atrial fibrillation ascertainment relied on scheduled Holter monitoring and prospective ECG-confirmed diagnoses, and asymptomatic or paroxysmal episodes may have been underdetected. However, given uniform assessment across participants, such misclassification is likely non-differential and would tend to attenuate associations. Stroke subtypes were not available; given their distinct pathophysiology, pooling heterogeneous phenotypes may have diluted associations with AtCM. Although internally validated, replication in independent and multiethnic cohorts is needed to confirm generalizability. Finally, translation into practice will require defining robust strategies to integrate structural, electrical, and autonomic markers across platforms and over time.

## Conclusion

Atrial cardiomyopathy is a cumulative, multidomain disease process in which structural, electrical, and autonomic abnormalities collectively define atrial disease burden. Longitudinal autonomic remodelling constitutes an independent and evolving axis within this framework, shifting the focus from isolated arrhythmia detection towards progressive characterization of atrial substrate.

## Supplementary Material

euag132_Supplementary_Data

## References

[1] Guichard J-B, Anselme F, Defaye P, Mansourati J, Pavin D, Pasquié J-L et al Prevention of atrial fibrillation after atrial flutter ablation with ramipril (from the PREFACE study). Am J Cardiol 2022;162:73–9. 34728062 10.1016/j.amjcard.2021.09.010

[2] Goette A, Corradi D, Dobrev D, Aguinaga L, Cabrera J-A, Chugh SS et al Atrial cardiomyopathy revisited—evolution of a concept: a clinical consensus statement of the European heart rhythm association (EHRA) of the ESC, the heart rhythm society (HRS), the Asian Pacific heart rhythm society (APHRS), and the Latin American heart rhythm society (LAHRS). Europace 2024;26:euae204. 39077825 10.1093/europace/euae204 PMC11431804

[3] Goette A, Kalman JM, Aguinaga L, Akar J, Cabrera JA, Chen SA et al EHRA/HRS/APHRS/SOLAECE expert consensus on atrial cardiomyopathies: definition, characterization, and clinical implication. Heart Rhythm 2017;14:e3–40. 27320515 10.1016/j.hrthm.2016.05.028 PMC5548137

[4] Guichard J-B, Nattel S. Atrial cardiomyopathy: a useful notion in cardiac disease management or a passing fad? J Am Coll Cardiol 2017;70:756–65. 28774383 10.1016/j.jacc.2017.06.033

[5] Chen P-S, Chen LS, Fishbein MC, Lin S-F, Nattel S. Role of the autonomic nervous system in atrial fibrillation: pathophysiology and therapy. Circ Res 2014;114:1500–15. 24763467 10.1161/CIRCRESAHA.114.303772 PMC4043633

[6] Guichard J-B, Hupin D, Pichot V, Berger M, Celle S, Borràs R et al Assessing heart rate fragmentation to predict atrial fibrillation in the general population aged 65 the PROOF-AF study. Eur Heart J Open 2025;5:oeaf030. doi: 10.1093/ehjopen/oeaf030. 40313732 PMC12042749

[7] Fyfe-Johnson AL, Muller CJ, Alonso A, Folsom AR, Gottesman RF, Rosamond W et al Heart rate variability and incident stroke: the atherosclerosis risk in communities study. Stroke 2016;47:1452–8. 27217501 10.1161/STROKEAHA.116.012662 PMC4880420

[8] Barthelemy J-C, Pichot V, Hupin D, Berger M, Celle S, Mouhli L et al Targeting autonomic nervous system as a biomarker of well-ageing in the prevention of stroke. Front Aging Neurosci 2022;14:969352. 36185479 10.3389/fnagi.2022.969352 PMC9521604

[9] Hillebrand S, Gast KB, de Mutsert R, Swenne CA, Jukema JW, Middeldorp S et al Heart rate variability and first cardiovascular event in populations without known cardiovascular disease: meta-analysis and dose–response meta-regression. Europace 2013;15:742–9. 23370966 10.1093/europace/eus341

[10] Hansen CS, Jørgensen ME, Malik M, Witte DR, Brunner EJ, Tabák AG et al Heart rate and heart rate variability changes are not related to future cardiovascular disease and death in people with and without dysglycemia: a downfall of risk markers? The whitehall II cohort study. Diabetes Care 2021;44:1012–9. 33526428 10.2337/dc20-2490 PMC7985416

[11] Jandackova VK, Scholes S, Britton A, Steptoe A. Are changes in heart rate variability in middle-aged and older people normative or caused by pathological conditions? Findings from a large population-based longitudinal cohort study. J Am Heart Assoc 2016;5:e002365. 26873682 10.1161/JAHA.115.002365 PMC4802439

[12] Barthélémy J-C, Pichot V, Dauphinot V, Celle S, Laurent B, Garcin A et al Autonomic nervous system activity and decline as prognostic indicators of cardiovascular and cerebrovascular events: the ‘PROOF’ study. Study design and population sample. Associations with sleep-related breathing disorders: the ‘SYNAPSE’ study. Neuroepidemiology 2007;29:18–28. 17898520 10.1159/000108914

[13] Heart rate variability: standards of measurement, physiological interpretation and clinical use. Task force of the European Society of Cardiology and the north American society of pacing and electrophysiology. Circulation 1996;93:1043–65. 8598068

[14] Pichot V, Roche F, Celle S, Barthélémy J-C, Chouchou F. HRVanalysis: a free software for analyzing cardiac autonomic activity. Front Physiol 2016;7:557. 27920726 10.3389/fphys.2016.00557 PMC5118625

[15] Costa MD, Davis RB, Goldberger AL. Heart rate fragmentation: a new approach to the analysis of cardiac interbeat interval dynamics. Front Physiol 2017;8:255. 28536533 10.3389/fphys.2017.00255 PMC5422439

[16] Bauer A, Kantelhardt JW, Barthel P, Schneider R, Mäkikallio T, Ulm K et al Deceleration capacity of heart rate as a predictor of mortality after myocardial infarction: cohort study. Lancet 2006;367:1674–81. 16714188 10.1016/S0140-6736(06)68735-7

[17] Guichard J-B, Guasch E, Roche F, Da Costa A, Mont L. Premature atrial contractions: a predictor of atrial fibrillation and a relevant marker of atrial cardiomyopathy. Front Physiol 2022;13:971691. 36353376 10.3389/fphys.2022.971691 PMC9638131

[18] Lang RM, Badano LP, Mor-Avi V, Afilalo J, Armstrong A, Ernande L et al Recommendations for cardiac chamber quantification by echocardiography in adults: an update from the American society of echocardiography and the European association of cardiovascular imaging. J Am Soc Echocardiogr 2015;28:1–39.e14. 25559473 10.1016/j.echo.2014.10.003

[19] 2024 ESC Guidelines for the management of atrial fibrillation developed in collaboration with the European Association for Cardio-Thoracic Surgery (EACTS)—PubMed. *Eur Heart J* 2024;45:3314–3414. doi:10.1093/eurheartj/ehae176. 10.1093/eurheartj/ehae176 39210723

[20] Moltó-Balado P, Reverté-Villarroya S, Monclús-Arasa C, Balado-Albiol MT, Baset-Martínez S, Carot-Domenech J et al Heart failure and Major adverse cardiovascular events in atrial fibrillation patients: a retrospective primary care cohort study. Biomedicines 2023;11:1825. 37509465 10.3390/biomedicines11071825 PMC10376826

[21] Vemulapalli S, Doros G, Weissler EH, Jones WS, Li S, Obrien SM et al Prediction of 1-year Major adverse cardiovascular events in chronic limb threatening ischemia. JACC Adv 2025;4:101959. 40695135 10.1016/j.jacadv.2025.101959 PMC12296431

[22] Johnson LS, Platonov PG, Conen D, Kennbäck C, Jujic A, Healey JS et al Markers of atrial myopathy in the general population: prevalence, predictors, and inter-relations. JACC Clin Electrophysiol 2023;9:2240–9. 37676201 10.1016/j.jacep.2023.07.012

[23] Johnson LSB, Juhlin T, Juul-Möller S, Hedblad B, Nilsson PM, Engström G. A prospective study of supraventricular activity and incidence of atrial fibrillation. Heart Rhythm 2015;12:1898–904. 25956964 10.1016/j.hrthm.2015.04.042

[24] Nattel S . Atrial cardiomyopathy manifestations in the general population. JACC Clin Electrophysiol 2023;9:2250–2. 38030333 10.1016/j.jacep.2023.09.019

[25] Goette A, Lemoine MD, Borof K, Schotten U, Breithardt G, Camm AJ et al Prevalence and severity of atrial cardiomyopathy in patients with recently diagnosed atrial fibrillation and stroke risk factors and its association with early rhythm control: a secondary analysis of EAST-AFNET 4. Europace 2025;27:euaf256. 41061672 10.1093/europace/euaf256 PMC12559886

[26] Habibi M, Chahal H, Greenland P, Guallar E, Lima JAC, Soliman EZ et al Resting heart rate, short-term heart rate variability and incident atrial fibrillation (from the multi-ethnic study of atherosclerosis (MESA)). Am J Cardiol 2019;124:1684–9. 31575421 10.1016/j.amjcard.2019.08.025 PMC6939867

[27] Agarwal SK, Norby FL, Whitsel EA, Soliman EZ, Chen LY, Loehr LR et al Cardiac autonomic dysfunction and incidence of atrial fibrillation: results from 20 years follow-up. J Am Coll Cardiol 2017;69:291–9. 28104071 10.1016/j.jacc.2016.10.059 PMC5260487

[28] Kim SH, Lim KR, Seo J-H, Ryu DR, Lee B-K, Cho B-R et al Higher heart rate variability as a predictor of atrial fibrillation in patients with hypertension. Sci Rep 2022;12:3702. 35260686 10.1038/s41598-022-07783-3 PMC8904557

[29] Schaarup JR, Bjerg L, Hansen CS, Grove EL, Andersen ST, Vistisen D et al Cardiovascular autonomic dysfunction precedes cardiovascular disease and all-cause mortality: 11-year follow-up in the ADDITION-PRO study. Diabetes Obes Metab 2025;27:5147–59. 40635178 10.1111/dom.16566 PMC12326908

[30] Baig M, Moafi-Madani M, Qureshi R, Roberts MB, Allison M, Manson JE et al Heart rate variability and the risk of heart failure and its subtypes in post-menopausal women: the women’s health initiative study. PLoS One 2022;17:e0276585. 36282885 10.1371/journal.pone.0276585 PMC9595519

[31] Costa MD, Redline S, Davis RB, Heckbert SR, Soliman EZ, Goldberger AL. Heart rate fragmentation as a novel biomarker of adverse cardiovascular events: the multi-ethnic study of atherosclerosis. Front Physiol 2018;9:1117. 30233384 10.3389/fphys.2018.01117 PMC6129761

[32] Guichard J-B, Guasch E. Preventing ischaemic stroke in patients with isolated sinus node disease: the value of evaluating atrial cardiomyopathy. Eur J Prev Cardiol 2024;31:504–6. 38085027 10.1093/eurjpc/zwad386

[33] Petek BJ, Al-Alusi MA, Moulson N, Grant AJ, Besson C, Guseh JS et al Consumer wearable health and fitness technology in cardiovascular medicine. JACC 2023;82:245–64. 37438010 10.1016/j.jacc.2023.04.054 PMC10662962

[34] Doehner W, Boriani G, Potpara T, Blomstrom-Lundqvist C, Passman R, Sposato LA et al Atrial fibrillation burden in clinical practice, research, and technology development: a clinical consensus statement of the European Society of Cardiology council on stroke and the European heart rhythm association. Europace 2025;27:euaf019. 40073206 10.1093/europace/euaf019 PMC11901050

