# Supplementary material for: Atrial cardiomyopathy as a multidomain disease: longitudinal evidence for autonomic remodelling
Source: Europace. 2026 Jun 30;28(6):euag132. doi: 10.1093/europace/euag132 (PMC13317983; doi:10.1093/europace/euag132)
Supplement: euag132_Supplementary_Data [file euag132_supplementary_data.docx]

# **Supplemental Material**

[Supplemental Material 0](#_Toc221175498)

[Methods 1](#_Toc221175499)

[**Clinical covariates** 1](#_Toc221175500)

[**Heart Rate Variability (HRV) assessment** 1](#_Toc221175501)

[**Expanded Statistical Analysis** 1](#_Toc221175502)

[Online Tables 5](#_Toc221175503)

[**Online Table 1. Comparison of Baseline Characteristics Between Included and Non-Included Participants** 5](#_Toc221175504)

[**Online Table 2. Baseline autonomic markers and incident atrial fibrillation.** 0](#_Toc221175505)

[**Online Table 3. ROC analysis and internal bootstrap validation of the static autonomic abnormalities construct** 0](#_Toc221175506)

[**Online Table 4. Longitudinal changes in autonomic markers and incident atrial fibrillation.** 0](#_Toc221175507)

[**Online Table 5.** **ROC analysis and internal bootstrap validation of the dynamic autonomic abnormalities construct** 0](#_Toc221175508)

[**Online Table 6. Incremental predictive performance of hierarchical models for incident atrial fibrillation.** 0](#_Toc221175509)

[Online Figures 1](#_Toc221175510)

[**Online Figure 1. Static autonomic abnormalities and incident atrial fibrillation** 1](#_Toc221175511)

[**Online Figure 2. Dynamic autonomic risk score and incident atrial fibrillation** 2](#_Toc221175512)

[**Online Figure 3. Cumulative HRV abnormalities and clinical markers of atrial cardiopathy** 3](#_Toc221175513)

## **Methods**

### **Clinical covariates**

Baseline clinical variables were obtained at study inclusion. Smoking status and alcohol consumption were self-reported. Obesity was defined as body mass index (BMI) >30 kg/m². Diabetes mellitus was defined as fasting plasma glucose ≥126 mg/dL or use of glucose-lowering medication. Hypertension was defined as mean systolic blood pressure ≥140 mmHg, mean diastolic blood pressure ≥90 mmHg (average of three measurements), or use of antihypertensive medication. Dyslipidemia and thyroid disorders were defined by current medication use. Severe obstructive sleep apnea was defined as an apnea–hypopnea index >30 events/hour assessed by home sleep study. Physical activity was assessed using the Population Physical Activity Questionnaire (POPAQ) and expressed as metabolic equivalent task hours per week (MET-h/week); physical inactivity was defined as <7.5 MET-h/week.¹⁵^,^¹⁷

### **Heart Rate Variability (HRV) assessment**

HRV parameters were derived from 24-hour Holter ECG recordings using validated automated algorithms following standard preprocessing, including artifact correction and exclusion of ectopic beats. Time-domain indices included pNN50, RMSSD, SDNN, and SDANN. Frequency-domain indices were computed using Fourier transform and included total power, very low frequency (VLF: 0.003–0.04 Hz), low frequency (LF: 0.04–0.15 Hz), and high frequency (HF: 0.15–0.40 Hz) components. Raw, logarithmic, relative values, and the LF/HF ratio were analyzed.

### **Expanded Statistical Analysis**

This section provides methodological details complementing the Statistical Analysis described in the main manuscript.

1. **Data presentation and preprocessing**

Continuous variables are reported as median and interquartile range (IQR), and categorical variables as counts and percentages. Frequency-domain HRV parameters exhibited right-skewed distributions and were log-transformed prior to analysis. HRV metrics were analyzed as continuous variables in all primary models.

1. **Longitudinal analysis of HRV parameters**

Within-individual changes in HRV between baseline (T0) and the 5-year follow-up visit (T5) were assessed in participants with paired measurements. Paired t-tests or Wilcoxon signed-rank tests were applied according to variable distribution. These analyses were descriptive and aimed to characterize autonomic remodeling over time.

1. **Prognostic analysis of static HRV parameters (baseline)**
   1. **Univariable Cox proportional hazards models**

Associations between baseline HRV parameters and incident atrial fibrillation (AF) were assessed using Cox proportional hazards models systematically adjusted for age, sex, and mean Holter heart rate. Given the limited number of incident AF events (n = 72), the number of covariates included in the models was intentionally restricted to preserve an adequate events-per-variable ratio and avoid model overfitting. Linearity was evaluated by comparing linear terms with natural cubic spline functions (3 degrees of freedom) using likelihood ratio tests. Effect estimates are reported as hazard ratios (HRs) with 95% confidence intervals, expressed as 75th versus 25th percentile contrasts. Proportional hazards assumptions were assessed using Schoenfeld residuals.

- 1. **Multivariable Cox modeling and variable selection**

HRV parameters associated with AF at p<0.10 in adjusted univariable analyses were considered for multivariable modeling. To limit collinearity and preserve biological interpretability, candidate variables were constrained by predefined HRV domains (time-domain, frequency-domain, fragmentation, and non-linear metrics), retaining the most informative parameter within each domain. Backward elimination based on the Wald statistic was applied, with p<0.05 required for retention. Non-linear terms identified in univariable analyses were preserved. This domain-based approach was defined a priori to balance parsimony and biological relevance.

- 1. **Construction of the static HRV score**

The static HRV score was defined as the linear predictor from the final multivariable Cox model, incorporating selected HRV parameters, with all models and scores adjusted for age, sex, and mean Holter heart rate. Discriminative performance was assessed using receiver operating characteristic (ROC) analysis. The optimal threshold was determined using the Youden index and internally validated by bootstrap resampling (1,000 iterations). Participants were dichotomized according to the validated cut-off, defining static HRV abnormality.

1. **Dynamic HRV analysis and landmark approach**

Dynamic autonomic remodeling was quantified as the change in HRV parameters between T0 and T5 (ΔHRV = T5 − T0). Associations between ΔHRV parameters and incident AF were evaluated using Cox models adjusted for age, sex, and change in mean Holter heart rate. To avoid immortal time bias, a landmark analysis was performed with time zero reset at the 5-year visit, excluding participants who developed AF prior to T5. A dynamic HRV score was derived as the linear predictor of the final model and dichotomized using ROC-derived thresholds.

1. **Cumulative effect of static and dynamic HRV abnormalities**

Participants were categorized into four groups: no abnormality, static abnormality only, dynamic abnormality only, or both abnormalities. AF-free survival was assessed using Kaplan–Meier estimates and compared with log-rank tests. Multivariable Cox models adjusted for age, sex, and mean Holter heart rate were used to estimate relative risks, with the no-abnormality group as reference. A secondary analysis grouped participants into three categories: no abnormality, either abnormality, or both abnormalities.

1. **Incremental prognostic value beyond conventional atrial cardiopathy markers**

Four nested prediction models were constructed: (M1) clinical variables; (M2) M1 plus electrical markers; (M3) M2 plus structural markers; and (M4) M3 plus autonomic (HRV) markers. Model discrimination was assessed using area under the ROC curve (AUC), with pairwise comparisons using the DeLong test. Incremental predictive performance was further evaluated using integrated discrimination improvement (IDI) and net reclassification improvement (NRI). Model parsimony was assessed using Akaike and Bayesian information criteria.

1. **Missing data, multiplicity, and software**

Analyses were conducted on complete cases without imputation. No formal adjustment for multiple comparisons was applied, as analyses were hypothesis-driven. Statistical analyses were performed using R version 4.5.0 (R Foundation for Statistical Computing).

## **Online Tables**

|  |  | **Included Patients  (n=670)** | **Non-included Patients  (n=341)** | P-value |  |
| --- | --- | --- | --- | --- | --- |
|  |  |  |  |  |  |
| ***Clinical features*** | |  |  |  |  |
|  | Age | 65.1 (65.0 - 66.1) | 65.1 (65.0 - 66.1) | 0.59 |  |
|  | Male gender | 276 (41.2%) | 126 (37.0%) | 0.22 |  |
|  | Diabetes mellitus | 38 (5.7%) | 19 (5.6%) | 0.99 |  |
|  | Hypertension | 240 (35.8%) | 125 (36.7%) | 0.85 |  |
|  | Dyslipidemia | 250 (37.4%) | 131 (38.4%) | 0.8 |  |
|  | Thyroid disorders | 103 (15.4%) | 47 (13.8%) | 0.56 |  |
|  | COPD | 30 (4.5%) | 21 (6.2%) | 0.33 |  |
|  | History of tobacco use | 235 (35.2%) | 129 (37.8%) | 0.45 |  |
|  | *Obesity* | *63 (9.4%)* | *45 (13.2%)* | *0.08* |  |
|  | Obstructive sleep apnea | 86 (12.8%) | 36 (10.6%) | 0.21 |  |
|  |  |  |  |  |  |
| ***Features Characterising Atrial Cardiopathy*** | | | | |  |
|  | PAC burden (per hour) | 2.4 (1.0 - 6.3) | 2.7 (1.2 - 6.2) | 0.25 |  |
|  | Increased PAC burden | 60 (9.0%) | 32 (9.4%) | 0.91 |  |
|  | Dilated left atrium | 116 (23.9%) | 5 (27.8%) | 0.78 |  |
|  | CHADS VA score | 3 (2 - 4) | 3 (2 - 4) | 0.28 |  |

### **Online Table 1. Comparison of Baseline Characteristics Between Included and Non-Included Participants**

Baseline clinical and atrial cardiopathy–related characteristics according to inclusion in the 5-year follow-up Holter ECG analysis. Patients without available follow-up Holter data were classified as non-included. Differences between groups were assessed to evaluate potential selection bias.

**Abbreviations**: CHADS-VA = Congestive heart failure, Hypertension, Age ≥75, Diabetes mellitus, Stroke/transient ischemic attack, Vascular disease, Age 65–74; COPD = chronic obstructive pulmonary disease; PAC = premature atrial contraction.

|  |  | Univariable Adjusted Model | | | | | |  | Multivariable Adjusted Model | | | | | |
| --- | --- | --- | --- | --- | --- | --- | --- | --- | --- | --- | --- | --- | --- | --- |
|  |  | aHR (Q3-Q2) | 95% CI | aHR (Q1-Q2) | 95% CI | P-value | Non-Linearity (p-value) |  | aHR (Q3-Q2) | 95% CI | aHR (Q1-Q2) | 95% CI | P-value | Non-Linearity (p-value) |
| ***Time-domain HRV Parameters*** | | |  |  |  |  |  |  |  |  |  |  |  |  |
|  | SDNN | 0.58 | (0.36 - 0.92) | 1.10 | (0.80 - 1.53) | 0.02 | No(0.10) |  | - | - | - | - | - | - |
|  | SDANN | 0.59 | (0.37 - 0.95) | 1.17 | (0.85 - 1.60) | 0.04 | No (0.25) |  |  |  |  |  |  |  |
|  | RMSSD | 1.01 | (0.73 - 1.40) | 0.93 | (0.72 - 1.21) | 0.93 | No (0.91) |  |  |  |  |  |  |  |
|  | pNN50 | 0.99 | (0.75 - 1.33) | 0.86 | (0.56 - 1.30) | 0.90 | No (0.78) |  |  |  |  |  |  |  |
| ***Frequency-domain HRV Parameters*** | | |  |  |  |  |  |  |  |  |  |  |  |  |
|  | Total power | 0.66 | (0.44 - 0.98) | 0.76 | (0.50 - 1.15) | 0.02 | Yes (0.03) |  |  |  |  |  |  |  |
|  | ULF | 0.90 | (0.56 - 1.43) | 0.70 | (0.47 - 1.05) | 0.09 | Borderline (0.06) |  |  |  |  |  |  |  |
|  | **VLF** | 0.81 | (0.48 - 1.35) | 0.77 | (0.52 - 1.16) | 0.003 | Yes (0.02) |  | 1.01 | (0.56 - 1.74) | 0.60 | (0.32 - 0.88) | 0.006 | Yes (0.004) |
|  | LF | 0.72 | (0.53 - 0.98) | 0.91 | (0.66 - 1.27) | 0.13 | No (0.20) |  |  |  |  |  |  |  |
|  | HF | 0.83 | (0.64 - 1.08) | 0.89 | (0.62 - 1.27) | 0.39 | No (0.22) |  |  |  |  |  |  |  |
|  | LF / HF | 1.26 | (0.81 - 1.95) | 1.18 | (0.95 - 1.48) | 0.17 | Borderline (0.09) |  |  |  |  |  |  |  |
| ***Advanced HRV Metrics*** | |  |  |  |  |  |  |  |  |  |  |  |  |  |
|  | PIP | 0.96 | (0.64 - 1.45) | 0.57 | (0.35 - 0.92) | 0.008 | Yes (0.02) |  | - | - | - | - | - | - |
|  | PSS | 0.94 | (0.65 - 1.38) | 0.66 | (0.41 - 1.08) | 0.03 | Borderline (0.05) |  |  |  |  |  |  |  |
|  | SD1 / SD2 | 1.14 | (0.82 - 1.60) | 0.97 | (0.81 - 1.16) | 0.89 | No (0.78) |  |  |  |  |  |  |  |
|  | α1 | 0.94 | (0.72 - 1.24) | 1.38 | (0.93 - 2.07) | 0.11 | No (0.16) |  |  |  |  |  |  |  |
|  | Sample Entropy | 1.06 | (0.72 - 1.56) | 0.94 | (0.69 - 1.27) | 0.98 | No (0.97) |  |  |  |  |  |  |  |
| ***Baroreflex-related autonomic markers*** | | | |  |  |  |  |  |  |  |  |  |  |  |
|  | Acceleration capacity | 1.10 | (0.58 - 2.07) | 0.70 | (0.49 - 1.00) | 0.07 | Yes (0.03) |  |  |  |  |  |  | - |
|  | **Deceleration capacity** | 0.70 | (0.42 - 1.15) | 1.02 | (0.71 1.46) | 0.03 | No (0.18) |  | 0.68 | (0.48-0.95) | 1.36 | (1.04 - 1.77) | 0.03 | No (0.24) |
|  |  |  |  |  |  |  |  |  |  |  |  |  |  |  |

### **Online Table 2. Baseline autonomic markers and incident atrial fibrillation.**

Associations between baseline HRV parameters and incident atrial fibrillation assessed using Cox proportional hazards models allowing for non-linear effects. Univariable models were adjusted for age, sex, and mean Holter heart rate. Hazard ratios are reported as quartile contrasts (Q3 vs Q2 and Q1 vs Q2). Non-linearity was assessed using likelihood-ratio tests comparing linear and natural cubic spline models. Multivariable results are derived from a parsimonious domain-based model including one representative HRV marker per autonomic domain.

Non-linearity p-value refers to the likelihood-ratio test comparing the linear versus cubic spline model for the same marker. Borderline non-linearity was defined as 0.05 ≤ p < 0.10.

Abbreviations: HRV : heart rate variability; ULF/VLF/LF/HF, ultra-/very-low-/low-/high-frequency power; PIP : percentage of inflection points; PSS : percentage of short segment ; AC/DC, acceleration/deceleration capacity.

| **Analysis** | **Cut-off** | **AUC** | **95%CI** | **Sensitivity** | **Specificity** | **PPV** | **NPV** | **Accuracy** |
| --- | --- | --- | --- | --- | --- | --- | --- | --- |
| **Training (Youden)** | 0.531 | 0.718 | 0.65–0.79 | 0.712 | 0.66 | 0.154 | 0.963 | 0.664 |
| **Bootstrap (median)** | 0.531 | 0.718 | 0.66–0.79 | 0.712 | 0.66 | 0.154 | 0.963 | 0.664 |

### **Online Table 3. ROC analysis and internal bootstrap validation of the static autonomic abnormalities construct**

ROC-derived performance metrics for the static autonomic abnormalities construct in the derivation sample and after internal bootstrap validation. The optimal cut-off was identified using the Youden index in the training sample and evaluated by bootstrap resampling.

Abbreviations : AUC : area under the curve; CI : confidence interval; PPV : positive predictive value; NPV : negative predictive value; ROC : receiver operating characteristic.

|  |  | Univariable Adjusted Model | | | | | |
| --- | --- | --- | --- | --- | --- | --- | --- |
|  |  | aHR (Q3-Q2) | 95% CI | aHR (Q1-Q2) | 95% CI | P-value | Non-Linearity (p-value) |
| ***Time-domain HRV Parameters*** | | |  |  |  |  |  |
|  | Δ SDNN | 0.92 | (0.64 - 1.31) | 0.98 | (0.73 - 1.33) | 0.19 | No(0.31) |
|  | Δ SDANN | 0.88 | (0.63 - 1.22) | 1.07 | (0.78 - 1.47) | 0.25 | No (0.26) |
|  | Δ RMSSD | 1.01 | (0.62 - 1.12) | 0.99 | (0.79 - 1.25) | 0.14 | No (0.91) |
|  | Δ pNN50 | 0.90 | (0.70 - 1.15) | 1.03 | (0.90 - 1.19) | 0.40 | No (0.35) |
| ***Frequency-domain HRV Parameters*** | | |  |  |  |  |  |
|  | Δ Total power | 0.97 | (0.66 - 1.42) | 0.81 | (0.58 - 1.14) | 0.03 | Yes (0.03) |
|  | Δ ULF | 1.07 | (0.82 - 1.40) | 0.85 | (0.66 - 1.10) | 0.28 | No (0.18) |
|  | Δ VLF | 0.98 | (0.67 - 1.45) | 0.84 | (0.60 - 1.18) | 0.05 | Yes (0.02) |
|  | Δ LF | 1.07 | (0.79 - 1.44) | 0.81 | (0.56 - 1.17) | 0.07 | Yes (0.03) |
|  | Δ HF | 0.93 | (0.79 - 1.09) | 1.06 | (0.93 - 1.20) | 0.51 | No (0.38) |
|  | Δ LF / HF | 1.05 | (0.85 - 1.30) | 0.97 | (0.71 - 1.32) | 0.91 | No (0.82) |
| ***Advanced HRV Metrics*** | |  |  |  |  |  |  |
|  | Δ PIP | 0.78 | (0.54 - 1.13) | 1.08 | (0.74 - 1.59) | 0.24 | No (0.19) |
|  | Δ PSS | 0.74 | (0.49 - 1.12) | 1.07 | (0.74 - 1.54) | 0.19 | No (0.12) |
|  | Δ SD1 / SD2 | 1.01 | (0.79 - 1.28) | 0.97 | (0.79 - 1.79) | 0.90 | No (0.83) |
|  | Δ α1 | 1.20 | (0.90 - 1.59) | 0.72 | (0.50 - 1.04) | 0.28 | No (0.15) |
|  | Δ Sample Entropy | 0.90 | (0.64 - 1.26) | 1.08 | (0.81 - 1.46) | 0.92 | No (0.93) |
| ***Baroreflex-related autonomic markers*** | | |  |  |  |  |  |
|  | Δ Acceleration capacity | 0.77 | (0.57 - 1.04) | 1.03 | (0.56 - 1.89) | 0.22 | No (0.12) |
|  | Δ Deceleration capacity | 0.98 | (0.67 - 1.42) | 0.87 | (0.60 - 1.26) | 0.29 | No (0.23) |
|  |  |  |  |  |  |  |  |

### **Online Table 4. Longitudinal changes in autonomic markers and incident atrial fibrillation.**

Associations between 5-year changes in heart rate variability (ΔHRV) parameters and incident atrial fibrillation assessed using Cox proportional hazards models allowing for non-linear effects. Models were adjusted for age, sex, and change in mean Holter heart rate. Hazard ratios are reported as quartile contrasts of within-individual change (Q3 vs Q2 and Q1 vs Q2). Non-linearity was evaluated using likelihood-ratio tests comparing linear and natural cubic spline models.

Non-linearity p-value refers to the likelihood-ratio test comparing the linear versus cubic spline model for the same marker. Borderline non-linearity was defined as 0.05 ≤ p < 0.10.

Abbreviations: HRV : heart rate variability; ULF/VLF/LF/HF, ultra-/very-low-/low-/high-frequency power; PIP : percentage of inflection points; PSS : percentage of short segment ; AC/DC, acceleration/deceleration capacity.

| **Analysis** | **Cut-off** | **AUC** | **95%CI** | **Sensitivity** | **Specificity** | **PPV** | **NPV** | **Accuracy** |
| --- | --- | --- | --- | --- | --- | --- | --- | --- |
| **Training (Youden)** | 0.477 | 0.667 | (0.59 -0.74) | 0.673 | 0.626 | 0.136 | 0.957 | 0.630 |
| **Bootstrap (median)** | 0.490 | 0.667 | (0.59 -0.74) | 0.635 | 0.647 | 0.135 | 0.953 | 0.646 |

### **Online Table 5.** **ROC analysis and internal bootstrap validation of the dynamic autonomic abnormalities construct**

ROC-derived performance metrics for the dynamic autonomic abnormalities construct in the derivation sample and after internal bootstrap validation. The optimal cut-off was identified using the Youden index in the training sample and evaluated by bootstrap resampling.

Abbreviations: AUC, area under the curve; CI, confidence interval; PPV, positive predictive value; NPV, negative predictive value; ROC, receiver operating characteristic.

| **Model** | **AUC (95% CI)** | **p-value vs.  previous model** | **p-value vs. Model 1** | **NRI** | **IDI** | **AIC** | **BIC** |
| --- | --- | --- | --- | --- | --- | --- | --- |
| **Model 1:** ***Clinical Risk Marker*** | 0.525 (0.448–0.602) | - | - | - | - | 365.99 | 374.94 |
| **Model 2:** Model 1 + ***Increased PAC Burden*** | 0.541 (0.464–0.619) | 0.289 | 0.289 | 0.17 | 0 | 365.48 | 374.43 |
| **Model 3:** Model 2 + ***Dilated LA*** | 0.570 (0.492–0.647) | 0.129 | **0.057** | 0.25 | 0 | 363.9 | 372.85 |
| **Model 4:** Model 3 + ***Abnormal HRV*** | 0.682 (0.606–0.750) | **<0.001** | **<0.001** | 0.74 | 0.02 | 351.03 | 359.98 |
|  |  |  |  |  |  |  |  |

### **Online Table 6. Incremental predictive performance of hierarchical models for incident atrial fibrillation.**

Stepwise comparison of nested prediction models sequentially incorporating clinical, electrical, structural, and autonomic domains. Discriminative performance was assessed using the area under the receiver operating characteristic curve (AUC) with 95% confidence intervals. Incremental model performance was evaluated using changes in AUC, net reclassification improvement (NRI), integrated discrimination improvement (IDI), and information criteria (AIC and BIC). P-values correspond to comparisons with the immediately preceding model and with the baseline clinical model.

**Abbreviations**: **AIC** = Akaike information criterion; **AUC** = area under the curve; **BIC** = Bayesian information criterion; **CI** = confidence interval; **HRV** = heart rate variability; **IDI** = integrated discrimination improvement; **LA** = left atrium; **NRI** = net reclassification improvement; **PAC** = premature atrial contraction.

## **Online Figures**

### **Online Figure 1. Static autonomic abnormalities and incident atrial fibrillation**

**Panel A.** Receiver operating characteristic (ROC) curve for the static autonomic abnormalities score, with an area under the curve (AUC) of 0.718. The optimal cut-off (0.531) was determined using the Youden index; shaded areas indicate 95% confidence intervals. **Panel B.** Kaplan–Meier curves for atrial fibrillation–free survival according to static autonomic abnormality status. The hazard ratio was estimated using Cox proportional hazards models adjusted for age, sex, and mean Holter heart rate. Numbers at risk are shown below the curves.

**Abbreviations:** AF, atrial fibrillation; AUC, area under the curve; CI, confidence interval; HR, hazard ratio; ROC, receiver operating characteristic.

### **Online Figure 2. Dynamic autonomic risk score and incident atrial fibrillation**

**Panel A.** Receiver operating characteristic (ROC) curve for the dynamic autonomic risk score predicting incident atrial fibrillation. The optimal cut-off was identified using the Youden index; the area under the curve (AUC) is shown. **Panel B.** Kaplan–Meier curves for atrial fibrillation–free survival according to dynamic autonomic abnormality status, defined using the Youden-optimized cut-off. Hazard ratios were estimated using Cox proportional hazards models adjusted for age, sex, and change in mean Holter heart rate. Numbers at risk are shown below the curves.

**Abbreviations:** AF, atrial fibrillation; AUC, area under the curve; CI, confidence interval; HR, hazard ratio; ROC, receiver operating characteristic.

### **Online Figure 3. Cumulative HRV abnormalities and clinical markers of atrial cardiopathy**

Comparison of clinical and structural AC markers across categories of cumulative HRV abnormalities. **Panel A:** CHADS-VA score. **Panel B:** prevalence of increased premature atrial contraction (PAC) burden. **Panel C:** prevalence of LA dilatation. Groups are defined according to the presence of no HRV abnormalities, either static or dynamic abnormalities, or both static and dynamic abnormalities.

**Abbreviations:** **HRV** = heart rate variability; **LA** = left atrium; **PAC** = premature atrial contraction; **PIP** = percentage of inflection points; **RMSSD** = root mean square of successive RR interval differences; **SDANN** = standard deviation of the average NN intervals in all 5-minute segments; **α1** = short-term fractal scaling
